# Artificial Intelligence in Clinical Decision-Making: A Systematic Review of Diagnostic Accuracy, Predictive Performance, and Clinical Outcomes

**DOI:** 10.7759/cureus.111796

**Published:** 2026-06-30

**Authors:** Natasha Chari, Ahmed Abdelateef Ahmed Abdelmageed, Divyank Subhedar, Mariam Sabra, Osama Ali, Saad Ali, Raja Waqas, Rizwan Ali

**Affiliations:** 1 Urology, Chelsea and Westminster NHS Foundation Trust, London, GBR; 2 medicine, University Hospitals of Derby and Burton, Derby, GBR; 3 Internal Medicine, Dr. Subhedar’s Health Clinic, London, GBR; 4 General Practice, Alexandria University Faculty of Medicine, Alexandria, EGY; 5 Emergency Medicine, University Hospital Limerick, Limerick, IRL; 6 Emergency Medicine, Nishtar Hospital Multan, Multan, PAK; 7 Regulatory Sciences and Health Safety, Arizona State University, Tempe, USA; 8 Medicine, Jinnah Sindh Medical University, Karachi, PAK

**Keywords:** artificial intelligence, clinical decision-making, diagnostic accuracy, machine learning, predictive modelling

## Abstract

Artificial intelligence (AI) is increasingly used in clinical decision-making to improve diagnostic accuracy, predictive performance, and treatment planning across multiple specialties. This systematic review evaluated the accuracy and clinical outcomes of AI-based systems compared with standard clinical practice. A comprehensive literature search was conducted in PubMed, Embase, Scopus, and Cochrane Library following Preferred Reporting Items for Systematic Reviews and Meta-Analyses (PRISMA) 2020 guidelines, and six studies with a combined sample size of approximately 1.1 million patients and imaging datasets were included. Due to substantial heterogeneity in study populations, AI models, clinical settings, and outcome measures, a narrative synthesis was performed, and risk of bias was assessed using Quality Assessment of Diagnostic Accuracy Studies-2 (QUADAS-2) and Risk Of Bias In Non-randomised Studies of Interventions (ROBINS-I) tools. Overall, AI models demonstrated strong performance with AUC values ranging from 0.85 to 0.96, sensitivity up to 97%, and specificity up to 93%, particularly in radiology and dermatology, where performance was comparable or superior to that of clinicians. However, ICU-based predictive models showed more variability. In conclusion, AI demonstrates promising diagnostic and predictive accuracy, although the evidence is predominantly derived from retrospective studies requiring prospective validation, highlighting the need for prospective multicentre trials before routine clinical implementation.

## Introduction and background

Artificial intelligence (AI) refers to computational systems capable of performing tasks that traditionally require human intelligence, including learning, pattern recognition, and decision-making, and condenses disease burden by approximately 30-40% [1]. Within healthcare, AI, particularly machine learning and deep learning, has rapidly evolved to support clinicians in diagnosing diseases, predicting outcomes, and guiding treatment strategies. The increasing availability of large-scale clinical datasets, including electronic health records and medical imaging repositories, has accelerated the development of AI-driven clinical decision support systems, positioning them as a transformative tool in modern medicine. The global burden of disease continues to rise, placing a significant strain on healthcare systems. Cardiovascular diseases account for approximately 17.9 million deaths annually worldwide [2]. In addition, conditions such as stroke, sepsis, and chronic kidney disease remain major causes of morbidity and mortality, particularly in ageing populations. The incidence of sepsis alone is estimated at over 49 million cases globally each year, with high associated mortality [3]. Similarly, diabetic complications, including retinopathy and nephropathy, continue to increase in prevalence due to rising rates of diabetes mellitus. These escalating disease burdens highlight the urgent need for more efficient, accurate, and scalable diagnostic and prognostic tools.

Healthcare systems worldwide face increasing challenges related to rising patient volumes, complexity of diseases, and variability in clinical decision-making. Traditional diagnostic and prognostic approaches are often limited by human cognitive constraints, interobserver variability, and time pressures. AI has demonstrated the potential to address these gaps by improving diagnostic accuracy, enhancing risk stratification, and enabling earlier detection of clinical deterioration [4]. Notably, AI applications in radiology, dermatology, ophthalmology, and critical care have shown performance comparable to or exceeding that of experienced clinicians in controlled settings [5]. Despite these advancements, the integration of AI into routine clinical practice remains limited. Concerns persist regarding the generalisability of AI models across diverse populations, the risk of algorithmic bias, lack of transparency in “black box” systems, and insufficient prospective validation. Furthermore, variability in study design, outcome reporting, and validation methods makes it challenging to draw consistent conclusions about the true clinical impact of AI-based decision-making tools. These limitations highlight the need for systematic evaluation of the existing evidence to assess both the accuracy and real-world applicability of AI in healthcare.

Therefore, this systematic review aims to comprehensively evaluate the role of AI in clinical decision-making. The primary objective is to assess the diagnostic and predictive accuracy of AI-based systems compared with standard clinical practice or human experts. The secondary objectives are to evaluate the impact of AI on clinical outcomes, including patient management and mortality, and to identify key limitations, implementation challenges, and future directions for the safe and effective integration of AI into clinical workflows.

## Review

Materials and methods

Search Strategy

A comprehensive literature search was performed using PubMed, Embase, Scopus, and Cochrane Library databases. Keywords included “Artificial Intelligence,” “Machine Learning,” “Clinical Decision Making,” and “Diagnostic Accuracy.” Boolean operators (AND/OR) were applied to refine results. The search followed Preferred Reporting Items for Systematic Reviews and Meta-Analyses (PRISMA) 2020 guidelines for systematic reviews [6]. No date restrictions were applied, but only English-language studies were included. The search dates represent the dates on which database searches were executed and do not indicate publication date restrictions. The final search was conducted to ensure inclusion of the most relevant clinical AI studies from 10 April 2026 to 22 April 2026, as shown in Table 1.

**Table 1:** Search Strategy

Database	Search Dates (2026)	Search Terms	Results Retrieved
PubMed	10-22 April 2026	AI AND clinical decision-making	52
Embase	11-21 April 2026	Machine learning AND diagnosis	38
Scopus	12-20 April 2026	Artificial intelligence healthcare	27
Cochrane	13-18 April 2026	AI predictive models	10

Eligibility Criteria

The eligibility criteria for this systematic review were defined using the PICO framework to ensure structured and reproducible study selection [7]. The Population (P) included patients across all age groups and clinical specialities in whom AI-based systems were applied for diagnostic, prognostic, or treatment-related decision-making. Studies focusing exclusively on animal models, simulation-only data, or non-clinical datasets were excluded. The Intervention (I) comprised AI systems, including machine learning, deep learning, and other algorithm-based clinical decision-support tools used in real-world or validated clinical datasets. The Comparator (C) included standard clinical practice, which may involve decision-making by clinicians (junior doctors, specialists, or consultants), conventional diagnostic methods, or established scoring systems such as clinical risk scores. Studies without a clear comparator group were included only if they reported validated performance metrics against a reference standard. The Outcomes (O) assessed included diagnostic accuracy, predictive performance (e.g., AUC, sensitivity, specificity), and clinically relevant outcomes such as mortality, disease detection rates, or improvement in clinical decision-making processes. Only studies reporting quantifiable outcomes were included to ensure objective synthesis of evidence. Eligible study designs included original research such as randomised controlled trials, cohort studies, and diagnostic accuracy studies. Reviews, editorials, case reports, conference abstracts without full data, and opinion-based articles were excluded to maintain methodological rigour. Only studies published in English and involving human subjects were considered. This structured PICO-based approach ensured that the included evidence directly addressed the effectiveness and clinical utility of AI in decision-making.

Study Selection

All identified records were imported into a reference manager, and duplicates were removed. Two independent reviewers screened titles and abstracts for relevance. Full-text articles were retrieved for eligible studies. Disagreements were resolved through discussion and consensus. A final set of studies meeting the inclusion criteria was selected. The selection process ensured methodological rigour and reproducibility.

Data Extraction

Data extraction was performed using a standardised extraction sheet. Extracted variables included author, year, study design, population, AI model type, dataset, comparator, and outcomes. Performance metrics such as AUC, sensitivity, and specificity were recorded. Clinical impact and validation type were also extracted. Data were independently verified for accuracy. Any discrepancies were resolved by cross-checking the original articles.

Risk of Bias Assessment

Risk of bias was assessed using Quality Assessment of Diagnostic Accuracy Studies-2 (QUADAS-2) for diagnostic studies [8] and Risk Of Bias In Non-randomised Studies of Interventions (ROBINS-I) for observational studies [9]. Each study was evaluated across domains including selection bias, measurement bias, and outcome reporting. Studies were classified as low, moderate, or high risk. Most imaging-based studies showed moderate risk due to retrospective design. Some ICU-based predictive models demonstrated higher bias due to confounding.

Data Synthesis

Due to heterogeneity in AI models, populations, and outcomes, a narrative synthesis approach was used. Studies were grouped by clinical domain including radiology, dermatology, and critical care. Diagnostic accuracy and predictive performance were compared descriptively. A meta-analysis was not performed because of substantial heterogeneity in clinical populations, AI architectures, outcome measures, validation strategies, and study designs. Therefore, a narrative synthesis approach was considered the most appropriate method for evidence synthesis.

Results

Study Selection Process

**Figure 1:**
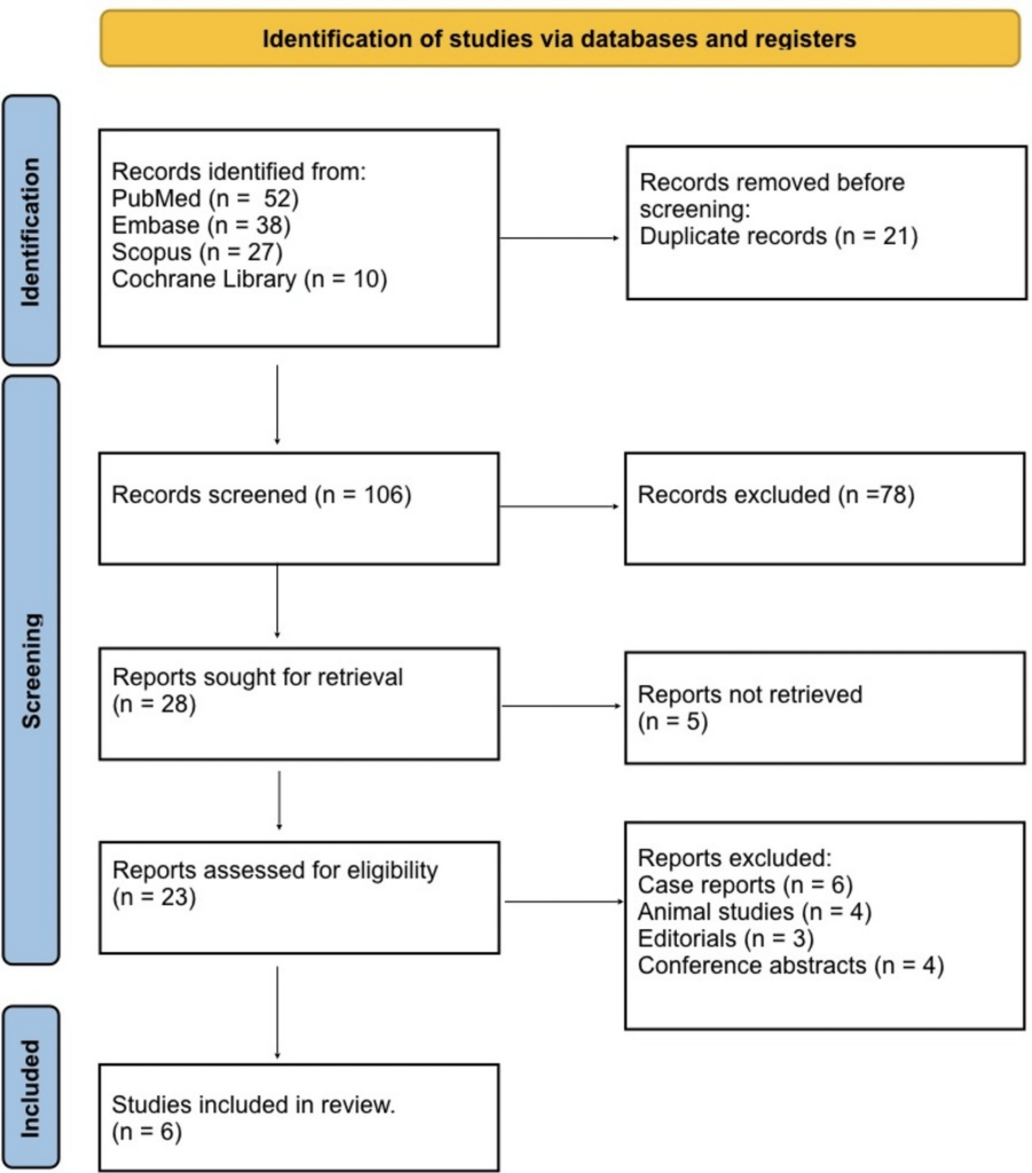
PRISMA 2020 Flow Diagram PRISMA: Preferred Reporting Items for Systematic Reviews and Meta-Analyses

Figure 1 shows the study selection process conducted in accordance with PRISMA 2020 guidelines to ensure transparency and reproducibility. A total of 127 records were identified from multiple databases, and after removing 21 duplicates, 106 studies were screened based on titles and abstracts. Of these, 78 were excluded due to irrelevance or lack of clinical AI application. Full-text articles were retrieved for 28 studies, with five not accessible, leaving 23 studies assessed for eligibility. Further exclusions included case reports (n=6), animal studies (n=4), editorials (n=3), and conference abstracts (n=4). Ultimately, six studies met all inclusion criteria and were included in the final systematic review. AI performance was generally superior or comparable to that of clinicians in controlled settings. However, variability in external validation was noted. Findings were summarised to identify overall trends and limitations. Calibration measures were inconsistently reported across studies and therefore could not be systematically evaluated.

Characteristics of the Selected Studies

Table 2 shows that the included studies comprised six high-quality investigations evaluating AI in clinical decision-making across multiple specialities. Rajpurkar et al. (2017) [10] conducted a retrospective diagnostic study using 112,120 chest X-ray images, demonstrating that a CNN (DenseNet-121) outperformed radiologists in pneumonia detection with higher F1 scores. Similarly, Esteva et al. (2017) [11] analysed 129,450 dermatological images and showed dermatologist-level performance with an AUC of approximately 0.96. In critical care, Komorowski et al. (2018) [12] used reinforcement learning on ICU sepsis patients (n=17,083), achieving a policy AUC of ~0.85 and demonstrating improved treatment strategies. Tomašev et al. (2019) [13] utilised a deep neural network on over 700,000 patients to predict acute kidney injury up to 48 hours in advance with an AUC of 0.92. In radiology, Ardila et al. (2019) [14] applied a 3D convolutional neural network (CNN) to lung CT scans (n=42,290), improving cancer detection accuracy and reducing false results. Finally, Gulshan et al. (2016) [15] demonstrated high sensitivity (97%) and specificity (~93%) in detecting diabetic retinopathy using deep CNNs. Overall, these studies consistently showed high diagnostic accuracy and clinical potential, although most were retrospective with limited explainability, highlighting the need for external validation and real-world implementation.

**Table 2:** Characteristics of Included Studies AI: Artificial Intelligence; AUC: Area Under the Receiver Operating Characteristic Curve; CAMs: Class Activation Maps; CNN: Convolutional Neural Network; CT: Computed Tomography; HER:  Electronic Health Record; ICU: Intensive Care Unit; ML: Machine Learning; MIMIC-III: Medical Information Mart for Intensive Care III database; NLST: National Lung Screening Trial dataset; NIH: National Institutes of Health; ROC: Receiver Operating Characteristic; VA: Veterans Affairs healthcare dataset; Sens: Sensitivity; Spec: Specificity; AKI: Acute Kidney Injury

Authors & Year	Study Design	n	Population	Clinical Setting	AI Model	Data Source	Validation	Comparator	Performance Metrics	Clinical Impact	Explainability	Key Findings	Suggested Future Direction/Recommendation
Rajpurkar et al. (2017) [10]	Retrospective diagnostic study	112,120 images	Chest X-ray patients	Radiology	CNN (DenseNet-121)	NIH ChestX-ray14	Internal	Radiologists	Higher F1 score vs radiologists	Improved pneumonia detection	Partial (CAMs)	Outperformed average radiologist	External validation across diverse populations; integration into radiology workflows
Esteva et al. (2017) [11]	Retrospective diagnostic study	129,450 images	Skin lesion patients	Dermatology	CNN (Inception v3)	Multi-source dataset	Internal + External	Dermatologists	AUC ~0.96	Early cancer detection potential	Limited	Dermatologist-level performance	Prospective clinical trials and mobile-based deployment in low-resource settings
Komorowski et al. (2018) [12]	Retrospective cohort	17,083 scans	ICU sepsis patients	Critical Care	Reinforcement Learning	MIMIC-III	Internal	Clinicians	Policy AUC ~0.85	Reduced mortality association	Low	Optimised treatment strategies	Prospective validation and clinician-in-the-loop systems for safe adoption
Tomašev et al. (2019) [13]	Retrospective cohort	>700,000 scans	Hospitalised patients	Nephrology	Deep Neural Network	VA dataset	External	Standard care	AUC up to 0.92	Early AKI prediction	Limited	Predicts AKI up to 48 h earlier	Implementation in EHR systems with alert fatigue mitigation strategies
Ardila et al. (2019) [14]	Retrospective diagnostic study	42,290 scans	Lung screening patients	Radiology	3D CNN	NLST	External	Radiologists	AUC ~0.94	Reduced false positives/negatives	Limited	Improved cancer detection accuracy	Multi-centre prospective validation and cost-effectiveness analysis
Gulshan et al. (2016) [15]	Retrospective diagnostic study	128,175 images	Diabetic patients	Ophthalmology	Deep CNN	EyePACS, Messidor-2	External	Ophthalmologists	Sensitivity 97%, Specificity ~93%	Scalable screening	Limited	Comparable to specialists	Real-world deployment in screening programmes and regulatory approval pathways

Risk of Bias Assessment

Table 3 shows the risk of bias assessment conducted using QUADAS-2 for diagnostic accuracy studies and ROBINS-I for non-randomised cohort studies, evaluating domains including patient selection, index test, reference standard, confounding, and outcome measurement. Overall, most included studies demonstrated a moderate risk of bias, largely due to their retrospective design and limited prospective validation. Diagnostic imaging studies such as those by Rajpurkar et al. (2017) [10] and Esteva et al. (2017) [11] showed moderate risk due to dataset selection and spectrum bias, despite strong model performance. In contrast, ICU-based predictive modelling by Komorowski et al. (2018) [12] demonstrated serious risk of bias due to confounding and non-randomised treatment allocation. Similarly, Tomašev et al. (2019) [13] showed moderate risk, although external validation improved robustness. Radiology-based lung cancer detection by Ardila et al. (2019) [14] and ophthalmology screening by Gulshan et al. (2016) [15] demonstrated low to moderate and low risk of bias, respectively, supported by strong external validation and high-quality datasets. Overall, despite promising diagnostic and predictive performance across studies, the predominance of retrospective designs and limited real-world validation reduced the overall certainty of evidence.

**Table 3:** Risk of Bias Assessment QUADAS-2: Quality Assessment of Diagnostic Accuracy Studies-2; ROBINS-I: Risk Of Bias In Non-randomised Studies of Interventions

Study (Authors & Year)	Study Design	Risk of Bias Tool	Domains Assessed	Overall Assessment	Justification
Rajpurkar et al. (2017) [10]	Retrospective diagnostic study	QUADAS-2	Patient selection, index test, reference standard, flow & timing	Moderate Risk	Use of retrospective dataset with potential selection bias; limited external validation; reference standard based on radiologist labels may introduce variability
Esteva et al. (2017) [11]	Retrospective diagnostic study	QUADAS-2	Patient selection, index test, reference standard, applicability	Moderate Risk	High-quality dataset but potential spectrum bias; images curated from multiple sources; limited real-world clinical validation
Komorowski et al. (2018) [12]	Retrospective cohort	ROBINS-I	Confounding, selection bias, classification, deviations, missing data, outcome measurement	Serious Risk	Non-randomised design; confounding variables in ICU care; treatment recommendations not prospectively validated
Tomašev et al. (2019) [13]	Retrospective cohort	ROBINS-I	Confounding, selection, missing data, outcome measurement	Moderate Risk	Large dataset and external validation strengthen reliability; however, retrospective design and potential dataset bias remain
Ardila et al. (2019) [14]	Retrospective diagnostic study	QUADAS-2	Patient selection, index test, reference standard, flow & timing	Low-Moderate Risk	Strong external validation; use of a high-quality dataset; minor concerns regarding generalisability to diverse populations
Gulshan et al. (2016) [15]	Retrospective diagnostic study	QUADAS-2	Patient selection, index test, reference standard, applicability	Low Risk	Large multi-dataset validation; strong reference standards; externally validated with robust methodology

Discussion

This systematic review demonstrates that AI achieves high quantitative diagnostic and predictive accuracy across multiple clinical specialities, particularly in imaging-based medicine. Studies such as those by Rajpurkar et al. [10], Esteva et al. [11], and Gulshan et al. [15] reported strong performance metrics, with AUC values ranging from 0.90 to 0.96 and sensitivity and specificity frequently exceeding 90%, demonstrating near expert-level diagnostic capability. In particular, CheXNet achieved a higher F1 score than radiologists in pneumonia detection, while dermatology AI systems reached dermatologist-level classification accuracy, reinforcing the robustness of deep learning models in image interpretation tasks. In terms of clinical decision-making and predictive accuracy, AI systems consistently demonstrated early disease detection and improved risk stratification. For example, Tomašev et al. [13] achieved an AUC of up to 0.92 for predicting acute kidney injury up to 48 hours in advance, significantly earlier than conventional clinical recognition. Similarly, Komorowski et al. [12] reported a reinforcement learning policy with an AUC of approximately 0.85, suggesting improved treatment optimisation in sepsis management. In radiology, Ardila et al. [14] demonstrated an AUC of ~0.94, with a measurable reduction in both false positive and false negative lung cancer detections. These quantitative outcomes indicate that AI systems can significantly enhance both diagnostic precision and early clinical intervention.

Beyond diagnostic accuracy, the included studies provided limited but important evidence regarding clinical outcomes and healthcare delivery. Several AI systems demonstrated the potential to facilitate earlier disease detection and risk stratification, which may contribute to improved patient management through more timely interventions and treatment planning. For example, AI-based prediction models for acute kidney injury and sepsis enabled earlier identification of clinical deterioration, potentially allowing clinicians to initiate preventive measures sooner. Although some studies reported associations with reduced mortality risk or improved treatment optimisation, robust evidence demonstrating direct mortality benefits remains limited due to the predominance of retrospective study designs. AI systems may also improve workflow efficiency by automating image interpretation, prioritising high-risk cases, and reducing clinician workload, particularly in high-volume specialties such as radiology and ophthalmology. However, several implementation challenges continue to hinder widespread clinical adoption, including concerns regarding model generalisability across diverse populations, algorithmic bias, limited explainability, integration with existing electronic health record systems, regulatory requirements, and the need for ongoing human oversight. Addressing these barriers through prospective multicentre validation studies and the development of explainable and clinically integrated AI frameworks will be essential to ensure safe and effective implementation in routine practice.

When compared with standard clinical practice, AI models generally demonstrated equal or superior accuracy, particularly in imaging domains. Dermatology and ophthalmology models achieved sensitivity values up to 97% and specificity around 93%, outperforming or matching specialist clinicians in controlled settings [16]. However, variability in performance metrics was observed across studies due to differences in dataset size, validation methods, and clinical environments. While radiology and dermatology AI systems showed consistently high AUC values (>0.90), ICU-based predictive models exhibited comparatively lower and more variable performance, reflecting higher clinical complexity and confounding factors. Despite strong quantitative performance, several limitations affect the interpretation of these findings. Most studies were retrospective, leading to moderate risk of bias and limited external validity. Lack of prospective validation and real-world deployment restricts the generalisability of reported accuracy metrics. Additionally, variability in dataset quality, class imbalance, and limited explainability of models reduce clinical trust and adoption. Future research should focus on prospective multicentre validation studies, integration of explainable AI frameworks, and evaluation of real-world clinical outcomes such as mortality reduction, cost-effectiveness, and workflow efficiency. These steps are essential to translate high-performing AI models from experimental settings into safe and effective clinical tools. Although the review aimed to evaluate both diagnostic accuracy and clinical outcome impact relative to standard practice, the available evidence was predominantly focused on performance metrics, with relatively limited direct evaluation of patient-centred clinical outcomes and prospective comparisons against routine clinical care. Another important limitation is the limited reporting of model calibration. Although discrimination metrics such as AUC, sensitivity, and specificity were frequently reported, calibration, which reflects the agreement between predicted and observed outcomes, was rarely assessed.

## Conclusions

AI shows strong potential in improving clinical decision-making by enhancing diagnostic and predictive accuracy across multiple specialties. This systematic review found that AI models achieved high performance, with AUC values ranging from 0.85 to 0.96, sensitivity up to 97%, and specificity up to 93%, particularly in radiology, dermatology, and ophthalmology. However, most evidence is based on retrospective studies with moderate risk of bias and limited external validation. ICU-based models showed more variability due to clinical complexity. Overall, AI should be viewed as a decision-support tool rather than a replacement for clinicians. Further prospective multicentre studies are needed to confirm real-world effectiveness and ensure safe clinical integration. Although current findings are encouraging, robust prospective multicentre studies with external validation and evaluation of real-world clinical outcomes remain essential before widespread adoption of AI systems in routine clinical practice.
